# Patient Acceptable Symptom State (PASS) Threshold in the Oxford Hip Score 12 Months Following Primary Total Hip Arthroplasty: Factors Associated with Failure to Achieve the Threshold and the Impact on Hip-Specific Outcomes, Quality of Life, and Satisfaction

**DOI:** 10.3390/jcm15166338

**Published:** 2026-08-17

**Authors:** Aiman Fatima, Anissa Hameed, Gillian Leitch, Nick D. Clement

**Affiliations:** 1 Medical School, Sunderland University, Sunderland SR1 3SD, UK; bh94zr@student.sunderland.ac.uk (A.H.); nickclement@doctors.org.uk (N.D.C.); 2 Edinburgh Orthopaedics, Royal Infirmary of Edinburgh, Edinburgh EH16 4SA, UK; gillian.leitch@nhs.scot

**Keywords:** Oxford hip score, PASS, hip arthroplasty

## Abstract

**Background:** The Oxford Hip Score (OHS) is a widely used hip-specific patient-reported outcome measure (PROM) following primary total hip arthroplasty (THA). This measure is calculated from answers to a 12-item questionnaire and can provide indices that indicate clinically meaningful success, such as patient acceptable symptom state (PASS). There were three purposes of the present single-cohort retrospective study: first, to determine the threshold PASS; second, to determine the influence of patient characteristics (age, gender, and body mass index (BMI)) and preoperative OHS and other PROMs of patients (namely, EQ-5D-3L, EQ-VAS, and pain VAS) who did not achieve this threshold; and third, to establish whether failure to achieve the PASS could still be associated with meaningful clinical improvement. **Methods:** This single-centre retrospective cohort study was conducted over 10 years from January 2013 to December 2022, during which 3997 patients completed pre- and postoperative OHS. The mean age was 68.3 years (standard deviation 11.7), and there were 2375 (59.4%) females in the cohort. Patient satisfaction was used as the anchor to define the PASS. **Results:** An OHS at 12 months postoperative of ≥36 points was identified as the threshold PASS (area under the curve 88.5%, 95% confidence interval [CI] 87.0 to 90.1, *p* < 0.001). Overall, 3002 (75.1%) achieved the PASS at 12 months. Lower BMI (odds ratio [OR] 0.96, 95% CI 0.95 to 0.98, *p* < 0.001) and higher preoperative OHS (OR 1.05, 95% CI 1.03 to 1.06, *p* < 0.001), EQ-5D (OR 1.88, 95% CI 1.33 to 2.65, *p* < 0.001) and EQ-VAS (OR 1.02, 95% 1.01 to 1.02, *p* < 0.001) were independently associated with achieving the PASS at 12 months. A total of 995 patients (24.9%) did not achieve this threshold. Patients not achieving the PASS had clinically meaningful worse (*p* < 0.001) postoperative outcomes and improvement relative to baseline in OHS, EQ-5D and EQ-VAS when compared to those achieving a PASS. Patients achieving the PASS were more likely to be satisfied with their THA (OR 16.8, 95% CI 12.8 to 22.1, *p* < 0.001); however, patients not achieving the PASS still had significant (*p* < 0.001) improvements relative to baseline in their OHS (9.6, 95% CI 9.0 to 10.2), EQ-5D (0.254, 95% CI 0.229 to 0.278), EQ-VAS (1.4, 95% CI −0.3 to 3.1) and pain VAS (8.2, 95% CI 6.1 to 10.3) and were more likely to be satisfied (70.8%) than not (29.2%). **Conclusions:** An OHS of ≥36 at 12 months was defined as the PASS. In total, 995 patients (29.4%) did not achieve this threshold, and in this group, patients were more likely to be female and have a high BMI and low preoperative OHS, EQ-5D, EQ-VAS, and pain VAS. Despite not achieving the PASS, they reported meaningful clinical improvements in OHS, EQ-5D, and pain VAS, and were satisfied with their THA (70.8%).

## 1. Introduction

There is widespread agreement that primary total hip arthroplasty (THA) provides symptomatic relief, restores joint functionality, and enhances quality of life; it was named the operation of the last century [1]. There are many hip-specific patient-reported outcome measures (PROMs) that are used as measures of the outcomes of THA [2,3]. The Oxford Hip Score (OHS) is an established PROM, evaluating pain and functional outcomes from the patient’s perspective [4,5]. Through this 12-item questionnaire, data interpretation can provide values reflecting clinically meaningful success [6]. An example is the patient acceptable symptom state (PASS) [7]. The PASS threshold is the minimum value of this parameter at which a patient considers their THA outcome as satisfactory.

Although THA is generally associated with substantial improvements in pain and function, implant-related biological responses may contribute to adverse outcomes in a proportion of patients. In particular, metallic wear debris and the release of metal ions can induce local biological reactions within the periprosthetic tissues, potentially contributing to inflammation, tissue damage and implant failure. A previous study published in *Diagnostics* highlighted the potential clinical consequences of metal debris and ion release following arthroplasty, including their association with adverse local tissue reactions and implant-related complications [8]. These findings emphasise that postoperative outcomes following THA are influenced not only by patient-reported symptoms and functional recovery but also by biological and implant-related factors.

The PASS was introduced to facilitate the interpretation of PROMs by identifying an absolute postoperative score beyond which patients perceive their symptoms as acceptable, rather than relying solely on the magnitude of improvement. In contrast to measures such as the minimal clinically important difference (MCID), which reflects the smallest change perceived as beneficial. PASS represents the symptom state achieved following treatment and is therefore considered particularly relevant when evaluating treatment success from the patient’s perspective. PASS thresholds are typically established using an external anchor, most commonly a global assessment of satisfaction or symptom acceptability and validated using receiver operating characteristic curve analysis or similar anchor-based approaches. While these methods have been widely adopted across orthopaedic outcome studies, differences in anchor selection, statistical methodology, follow-up duration, and study population can influence the estimated threshold and may limit the generalisability of reported PASS values between healthcare settings.

The currently defined PASS thresholds for the OHS range between 30.6 and 40.5 points following THA, with recent literature citing the mean PASS threshold for a Scottish patient to be 38.3 at 12 months postoperative [9,10,11]. This wide variability is because the threshold is influenced by myriad factors, such as patient age and gender, cohort size, the postoperative time at which the threshold was determined, the method used to determine the PASS, and the surgical approach [7,8,9,10,11,12,13,14,15,16].

Numerous patient characteristics influence preoperative OHS, with examples being age, gender, body mass index (BMI), educational achievement, psychometric conditions (for example, depression, anxiety, and low expectations) and ASA grade [17,18,19]. There are several aspects of the PASS threshold that, to date, have received scant research attention. Among them are the influence of patient characteristics on achievement of the PASS threshold and the association between achievement of the PASS threshold, on the one hand, and high levels of patient functional improvement and quality of life, on the other, and the discriminative value of the threshold (and hence its clinical utility).

There were three purposes of the present single-centre retrospective cohort study: first, to determine the PASS threshold in the OHS 12 months postoperative; second, for patients who do not achieve the threshold, to determine the influence of their characteristics on the threshold; and third, for these patients, to determine the nature of the differences between pre- and postoperative levels of outcome measures.

## 2. Materials and Methods

Ethical approval was obtained for data collection and analysis (Scotland B REC, number 20/SS/0125 A, approved on 10 December 2020) and was conducted in accordance with the Declaration of Helsinki and the guidelines for good clinical practice [20]. This single-centre retrospective cohort study was conducted over 10 years from January 2013 to December 2022. The study centre has an established arthroplasty register into which prospective patient demographic data and pre- and postoperative PROMS are entered. Patients who underwent primary THA for arthritis completed a preoperative questionnaire including demographics and PROMS, which were also repeated 12-months postoperatively via a postal questionnaire. Patients who did not return the questionnaire or had missing responses to specific questions were routinely contacted via telephone to complete it. This is a valid way to collect the OHS [21].

Eligible participants were identified from our prospectively maintained institutional arthroplasty registry. All consecutive adult patients who underwent primary THA during the study period and completed both preoperative and 12-month postoperative patient-reported outcome measures were eligible for inclusion. Patients were excluded if they underwent revision THA, hip resurfacing, or arthroplasty for acute trauma, had incomplete or missing outcome data required for PASS determination, or were lost to follow-up before the 12-month assessment. Where patients underwent bilateral THA during the study period, only the first eligible procedure was included to maintain statistical independence.

The responses to each of the OHS questions were scored from 0 to 4 [5,22]. A summative score of 48 is the best possible score (least symptomatic), and 0 is the worst possible score (most symptomatic). HRQoL was assessed using the EQ-5D-3L questionnaire, with the responses recorded at three levels (3L) [23]. These range from −0.594 (worst health) to 1 (best health). The EQ visual analogue scale (VAS) for general health was also used, with the scale ranging from 0 (worst) to 100 (best) [23]. The MCID in the OHS was defined as five points [24], 0.085 for the EQ-5D and 6.4 for the EQ-VAS [25]. Patients were also asked to complete a pain VAS, where 0 was the worst pain and 100 was no pain.

Patients were asked at 12 months how satisfied they were with their THA, with responses recorded on a 5-point Likert scale: very satisfied (5), satisfied (4), neutral (3), dissatisfied (2), or very dissatisfied (1). Patients satisfied or very satisfied were defined as satisfied, and those answering neutral, dissatisfied, or very dissatisfied were categorised as dissatisfied [26]. The PASS was defined using patient satisfaction as the anchor question. The threshold (score) in the postoperative OHS that offered the greatest predictive value for patient satisfaction at 12 months was used to determine the optimal value.

## 3. Statistical Analysis

Statistical analysis was performed using Statistical Package for Social Sciences version 17.0 (SPSS Inc., Chicago, IL, USA). Simple descriptive analysis was undertaken according to mean and standard deviation (SD) for pre- and postoperative PROMs. The distribution of continuous variables was assessed using visual inspection of histograms and Q–Q plots, together with the Shapiro–Wilk test for normality. As continuous variables were considered approximately normally distributed, parametric statistical tests were used. Paired and independent Student’s *t*-tests were used to compare continuous variables within and between groups, respectively. A chi-square test was used to compare categorical variables between groups. Receiver operating characteristic (ROC) curve analysis was used to identify a threshold (point of maximal sensitivity and specificity) in the mean postoperative OHS to define the PASS. This is reported as an area under the ROC curve (AUC), where 0.5 equates to no discrimination, 0.5–0.7 indicates poor discrimination, 0.7–0.8 acceptable discrimination, 0.8–0.9 excellent discrimination, and >0.9 outstanding discrimination [27].

To identify factors associated with achieving the PASS threshold at 12 months, univariable logistic regression analyses were first performed for each candidate preoperative variable. Variables demonstrating statistical significance on univariable analysis (*p* < 0.05), together with clinically relevant variables identified a priori, were subsequently entered into a multivariable logistic regression model to identify independent predictors of PASS achievement. Adjusted odds ratios (ORs) with 95% confidence intervals (CIs) are reported. Statistical significance was defined as a two-sided *p* < 0.05. Given that the analyses were hypothesis-driven and aimed to evaluate prespecified associations rather than to undertake exploratory multiple testing, a Bonferroni correction was not applied, as this may have increased the risk of Type II error and obscured clinically meaningful associations.

As this was a retrospective observational study based on a prospectively maintained institutional registry, no a priori sample size calculation was performed. Instead, the sample size was determined by all eligible patients who met the predefined inclusion criteria during the study period. The large available cohort was considered sufficient to provide robust estimates of the PASS threshold and to support the planned regression analyses.

## 4. Results

During the study period, 3997 patients completed their postoperative OHS at 12 months. In this cohort, the mean age was 68.3 years (standard deviation [SD] 11.7, range 24 to 96 years), and 2375 females (59.4%) completed the postoperative OHS at 12 months. The mean preoperative OHS for the total cohort was 20.9 (SD 9.0), which improved to 39.6 (SD 9.1) at 12 months postoperatively. The difference in these scores was significant (mean difference 18.7, 95% CI 18.4 to 19.1, *p* < 0.001, paired *t*-test).

**Figure 1:**
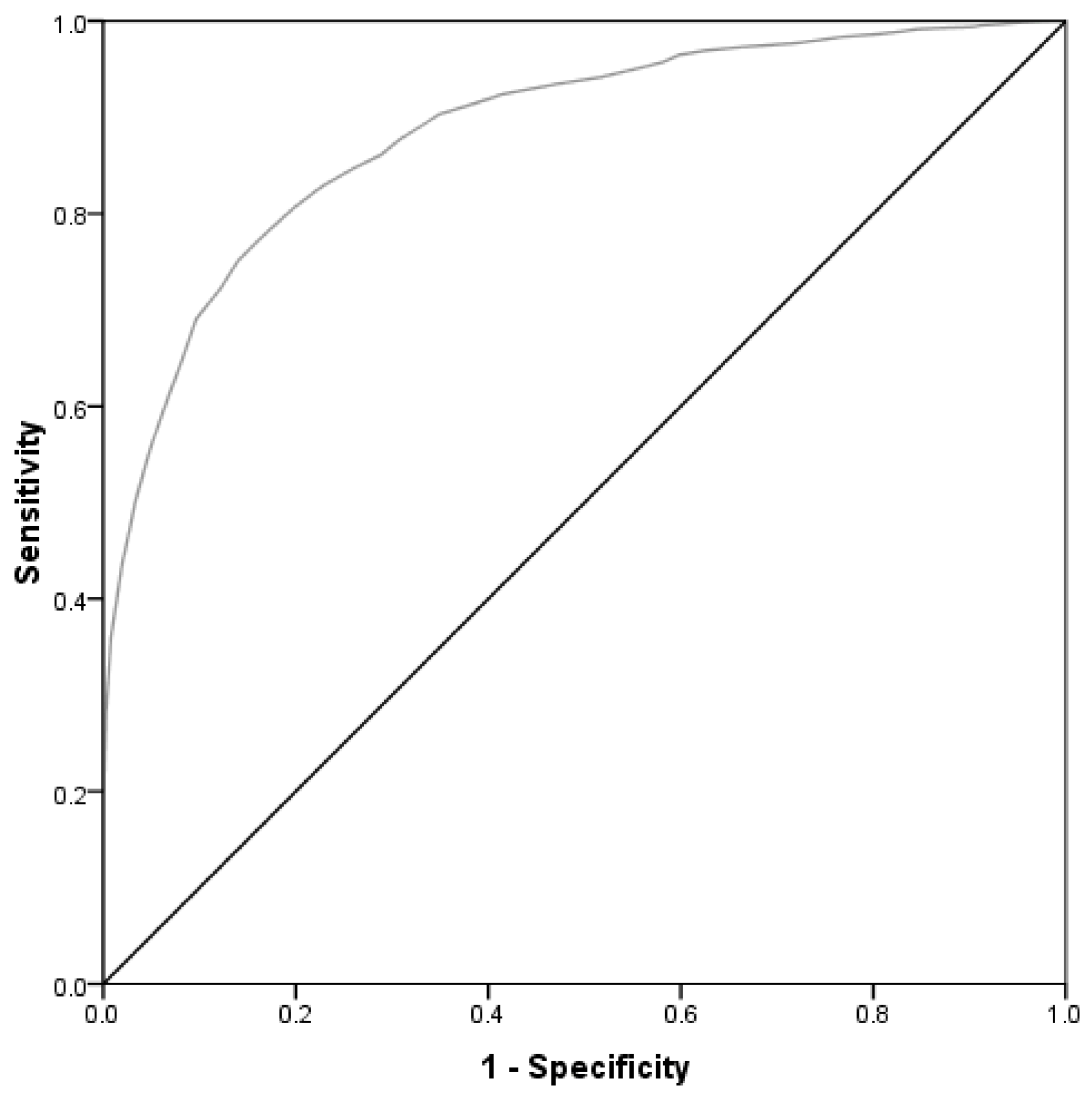
Receiver operating characteristic curve for patient satisfaction with their hip arthroplasty at 12 months using the postoperative Oxford Hip Score (OHS). The area under the curve was 88.5% (95% CI 87.0 to 90.1, *p* < 0.001).

**Figure 2:**
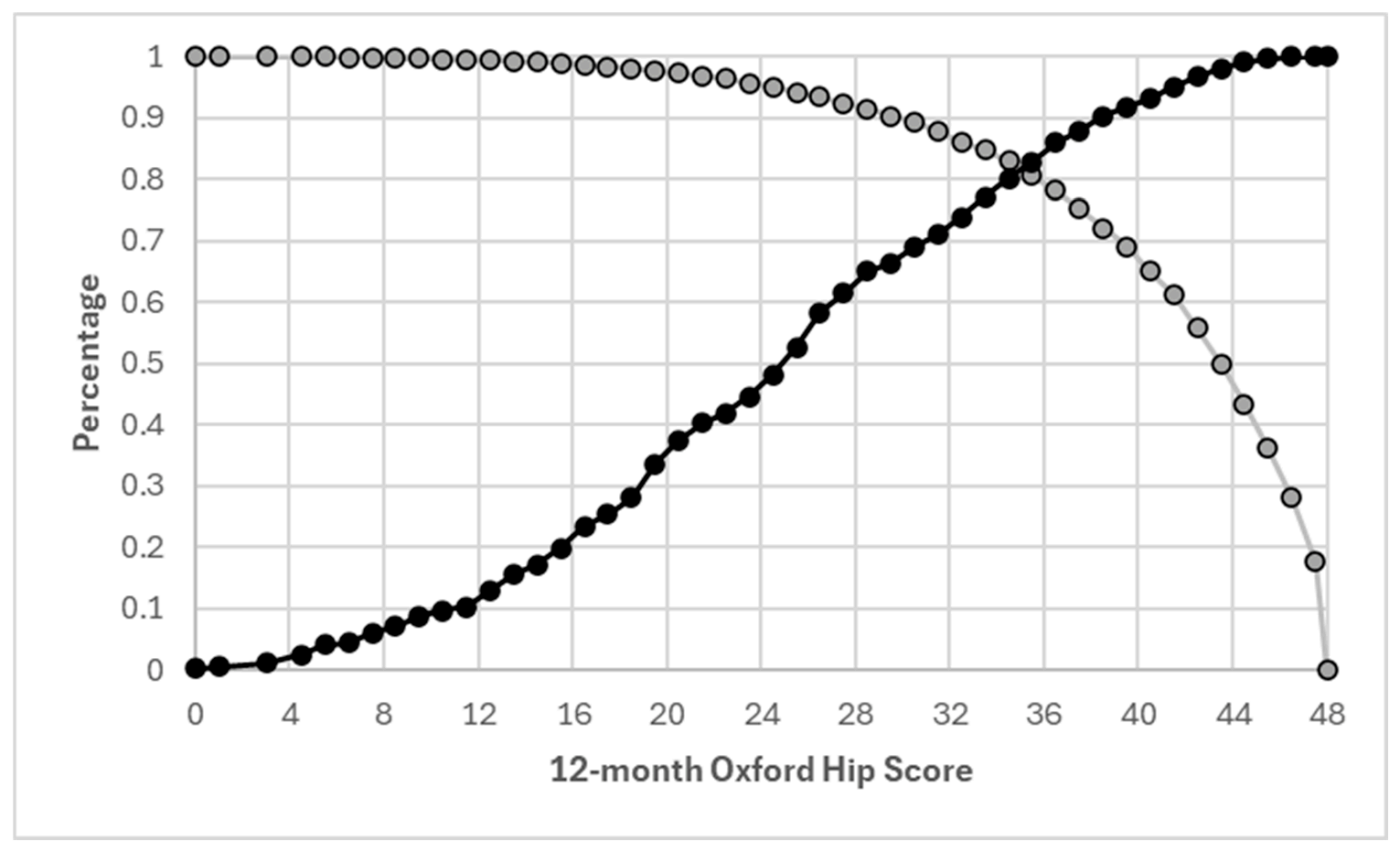
Sensitivity (grey) and specificity (black) plots for predicting patient satisfaction according to their 12-month Oxford Hip Score.

The threshold value in the 12-month OHS that offered maximal sensitivity and specificity was 36 points and had an AUC of 88.5% (95% CI 87.0 to 90.1, *p* < 0.001), being an excellent discriminator (Figure 1 and Figure 2). Therefore, the PASS was defined as an OHS of 36 points or more.

**Figure 3:**
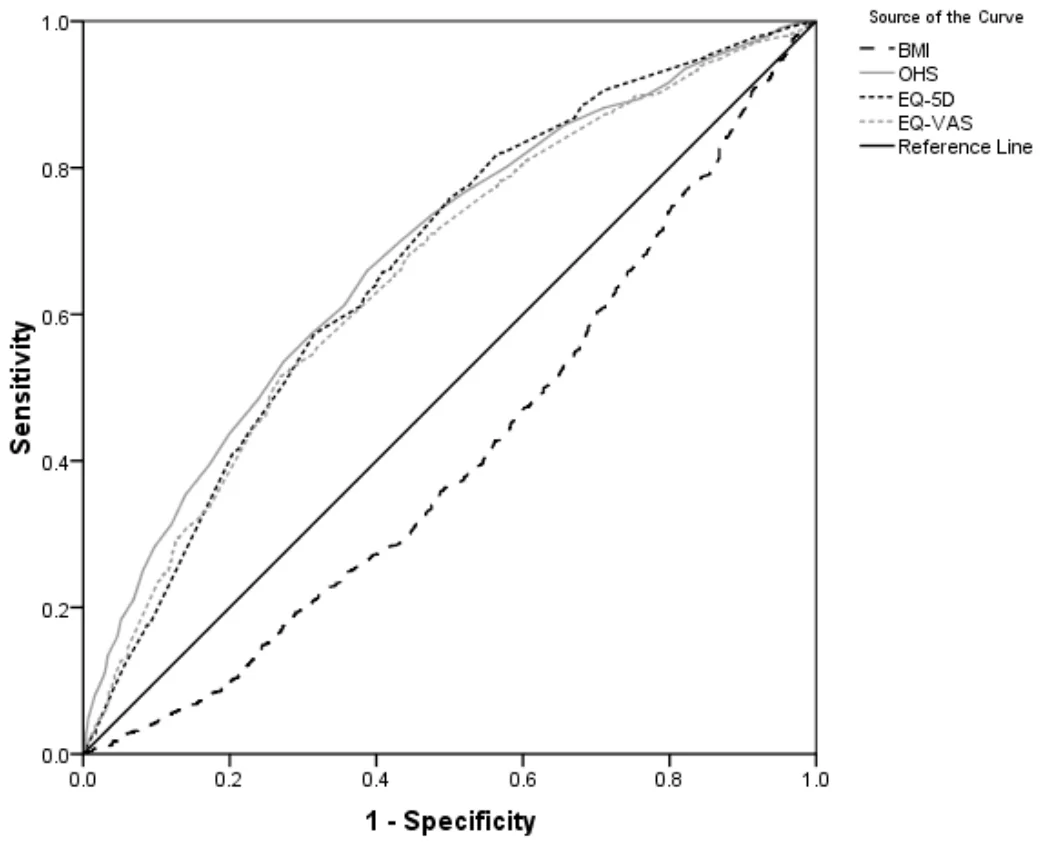
Receiver operating characteristic curve for patient satisfaction with their total hip arthroplasty at 12 months using the preoperative body mass index (BMI), Oxford Hip Score (OHS), EQ-5D and EQ-VAS. The area under the curve was 70.7% (95% CI 68.8 to 72.6, *p* < 0.001).

**Figure 4:**
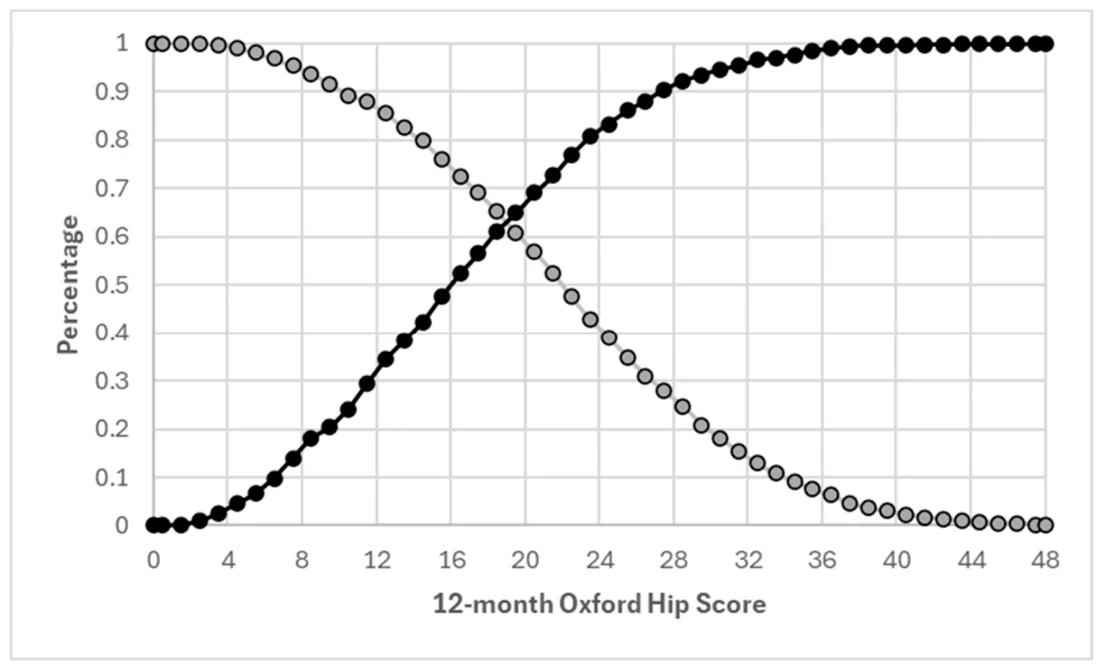
Sensitivity (grey) and specificity (black) plots for predicting achievement of a patient acceptable symptom state according to the preoperative Oxford Hip Score.

There were 3002 patients (75.1%) who achieved the PASS at 12 months and 995 patients (24.9%) did not. Characteristics of the latter group were more likely to be female (*p* = 0.001), had a higher BMI (*p* < 0.001) and lower preoperative OHS (*p* < 0.001), EQ-5D (*p* < 0.001), EQ-VAS (*p* < 0.001), and pain VAS (*p* < 0.001) (Table 1 and Table 2). When adjusting for confounding factors, a lower BMI (odds ratio [OR] 0.96, 95% CI 0.95 to 0.98, *p* < 0.001) and higher preoperative OHS (OR 1.05, 95% CI 1.03 to 1.06, *p* < 0.001), EQ-5D (OR 1.88, 95% CI 1.33 to 2.65, *p* < 0.001) and EQ-VAS (OR 1.02, 95% 1.01 to 1.02, *p* < 0.001) were independently associated with achieving a PASS at 12 months following THA (Table 2). When assessing the association with achieving a PASS of these independent variables, the OHS had the greatest AUC (70.0%, 95% CI 66.1 to 74.0) when compared to BMI (58.7%, 95% CI 56.3 to 61.1), EQ-5D (66.9%, 95% CI 64.6 to 69.1) and EQ-VAS (65.7%, 95% CI 63.5 to 68.0). A threshold value of 19 points in the preoperative OHS offered the maximal sensitivity and specificity and was an acceptable discriminator (Figure 3 and Figure 4). Therefore, patients with a preoperative OHS of less than 19 were less likely to achieve a postoperative PASS at 12 months following THA.

Patients not achieving a PASS had clinically meaningful worse postoperative outcomes and improvement in their OHS and EQ-5D and EQ-VAS when compared to those who achieved the PASS (Table 3). Although those patients who did not achieve the PASS had worse outcomes, the improvements in their OHS and EQ-5D between preoperative and postoperative at 12 months were both clinically significant (beyond the MCID) and statistically significant (Table 3). Patients who achieved the PASS at 12 months were likely to be more satisfied with their THA than those who did not achieve the PASS (OR 16.8, 95% CI 12.8 to 22.1, chi-square) (Table 4). However, despite this difference, patients who did not achieve the PASS threshold at 12 months postoperative were still more likely to be satisfied (70.8%) than not (29.2%) (Table 4).

Among patients who did not achieve the PASS threshold for the OHS at 12 months following THA, there were no significant differences between those who were satisfied and those who were not satisfied with surgery in terms of sex, age, BMI, preoperative EQ-5D, EQ-VAS, or pain VAS scores (all *p* > 0.05). However, patients who reported satisfaction had significantly lower preoperative OHS scores than those who were not satisfied (mean 16.1 vs. 17.9; mean difference 1.8, 95% CI 0.7 to 2.9; *p* = 0.002), indicating poorer preoperative hip-specific function among satisfied patients (Table 5).

## 5. Discussion

This study has shown that the PASS threshold in the 12-month OHS was ≥36 according to patient satisfaction, which was an excellent discriminator according to the AUC. Patients not achieving PASS were more likely to be female, had a higher BMI, and had lower baseline scores (OHS, EQ-5D, EQ-VAS, pain VAS). The preoperative OHS, with a threshold of 19 points or more, was an acceptable predictor of reaching a postoperative PASS. Although those not achieving the PASS had worse outcomes, they still experienced MCIDs in OHS and EQ-5D. Satisfaction with THA was more likely in those achieving the PASS, but 70.8% of those not achieving the PASS still reported being satisfied with their outcome.

The predictive thresholds for the postoperative PASS in the OHS vary considerably depending on methodology, anchors, and patient demographics. Harris et al. conducted a prospective cohort study of 706 patients undergoing THA, establishing a PASS threshold of 30.6 on the OHS using anchor-based predictive modelling with parametric logistic regression [9]. This method, which adjusted for anchor reliability and response bias, produced narrower confidence intervals and a more precise cut-off than earlier ROC-based studies. The derived threshold corresponded with a high observed satisfaction of 88.4% at 12 months. ROC analysis employed in the current study identified a slightly higher PASS threshold of 36 points, with a similar satisfaction rate of 90.9%. In contrast, a large Dutch retrospective analysis of 40,213 primary THAs found a higher OHS PASS threshold of ≥40.5 [10]. This study applied the Youden method following ROC analysis, which aims to optimise sensitivity and specificity, which may have elevated the cut-off. The current study placed satisfaction as an anchor, whereas the Dutch study manufactured an anchor that demanded both a precise and high level of functional change; this selectivity may have further increased the threshold. Their study comprised a higher proportion of females (63.8%), and most participants were between 70–79 years, which may have influenced the PASS threshold. Therefore, the influence of methodological variance, population demographics and external anchor selection in influencing cut-offs should be considered when interpreting PASS attainment [10].

Identifying patients at risk of not achieving a PASS allows for more realistic expectation-setting and potential adjustment of perioperative management. Multiple studies demonstrate that higher preoperative scores predict better postoperative outcomes [18,28]. A prospective single-centre study of 120 patients found that the preoperative OHS was a consistent predictor of outcomes at 3, 6, and 12 months, while lower baseline scores were strongly associated with poorer postoperative attainment [19]. The current study found that both lower OHS and HRQoL were independently associated with failure to achieve a PASS. This suggests that those with the worst baseline function, such as those waiting longer for their surgery [29], are less likely to be satisfied with the outcome of their THA.

Female patients are consistently less likely to achieve a PASS compared with males [30]. A prospective study of 1120 THA patients reported that, at 1 year, women walked shorter distances, required more assistance, and reported more pain on walking than men [30]. Anatomical and biomechanical factors are thought to contribute to differences in outcomes following THA [31]. A retrospective study highlighted predictors of poorer outcomes within male and female groups, respectively, suggesting gender-adjusted PASS scores may lead to better predictions of contentment [32]. There was also no independent effect of gender on the risk of achieving a postoperative PASS. However, females [33] and those with a higher BMI [34] have a lower preoperative baseline functional score, which is associated with a failure to achieve a PASS.

The role of BMI is debated. Traditional analyses found that a higher BMI is associated with poorer outcomes. For example, a pooled analysis of four large cohorts showed every five-unit increase in BMI corresponded to a 0.78-point reduction in 12-month OHS, although improvements remained substantial across all groups [19]. More recent evidence challenges this, with a prospective observational study (n = 1736) reporting greater pain reduction in obese patients and higher satisfaction in Class II obesity compared with normal BMI [35]. However, this is in contrast to the present study, where a higher BMI was independently associated with not achieving a postoperative PASS, despite the potentially larger absolute gains from poorer baseline status.

In the current cohort, although 24.9% of the patients did not achieve the PASS, 70.8% of them reported being satisfied with their outcome, highlighting the presence of a “satisfied non-achieving” subgroup. These discrepancies raise concerns about over-reliance on rigid thresholds, which may misclassify patient experiences and underestimate success. Nevertheless, patients failing to achieve the PASS reported worse outcomes. In the present study, non-responders achieved smaller functional gains and lower postoperative OHS scores. Similarly, a multicentre cohort study found that non-achievers were less satisfied and less willing to undergo surgery again [7]. A retrospective analysis using the WOMAC score also demonstrated that non-achievers experienced worse stiffness, pain, and function [36]. These findings confirm that while some patients are satisfied despite not reaching a PASS, those failing to achieve this threshold may face a more adverse functional trajectory.

Patient satisfaction was selected as the external anchor because it represents the patient’s global assessment of treatment success and has been widely used in previous studies to establish PASS thresholds following THA and other orthopaedic procedures. This approach provides methodological consistency with the existing literature and facilitates comparison across studies. However, satisfaction and symptom acceptability are related but conceptually distinct constructs. Whereas a PASS reflects whether patients consider their current symptom state acceptable, satisfaction may also be influenced by factors beyond symptom severity, including preoperative expectations, the care experience, communication with healthcare providers, and perioperative complications. Consequently, the PASS threshold derived using satisfaction as the anchor may be influenced by these broader aspects of the patient experience. Despite this limitation, the strong association observed between achieving the PASS and patient satisfaction in the present study supports the validity of satisfaction as a clinically relevant external anchor.

Although the multivariable logistic regression model was statistically significant and demonstrated acceptable calibration, its explanatory performance was modest, accounting for only 11–16% of the variance in achieving the PASS. This suggests that while higher preoperative BMI, lower OHS, lower EQ-5D and lower EQ-VAS scores were independently associated with failure to achieve an acceptable symptom state, these factors alone have limited ability to predict individual patient outcomes. The relatively low proportion of explained variance indicates that other important determinants of postoperative symptom acceptability were not captured within the registry. These may include psychological factors, patient expectations, comorbidities, surgical technique, implant-related factors, postoperative rehabilitation, and the occurrence of complications. Future studies incorporating these variables may improve prediction models and provide a more comprehensive understanding of the factors influencing achievement of the PASS following THA.

There were several limitations to this retrospective study that should be acknowledged. The current study reported the cohort as a combined single group, and, potentially, the responses to the individual questions may be different according to patient demographics, such as age and gender, for example [37]. The study did not include additional patient variables, such as comorbidity, which has been demonstrated to be associated with lower rates of postoperative patient satisfaction with their arthroplasty [18,38,39,40]. This may in part account for the fact that the OHS questions only predicted up to 16% of the variation in the model, with the remainder being due to other variables. A further limitation is that comprehensive comorbidity data were not available within our institutional arthroplasty registry and therefore could not be included in the analyses. Comorbid medical conditions, as well as other unmeasured factors such as mental health status, patient expectations, and socioeconomic characteristics, may influence postoperative symptom perception, satisfaction, and the likelihood of achieving the PASS threshold. Consequently, residual confounding cannot be excluded, and the observed associations should be interpreted within the context of these unmeasured variables.

In conclusion, an OHS of ≥36 at 12 months was defined as the PASS after THA and was associated with significantly higher patient satisfaction. Lower preoperative OHS and EQ-VAS scores were associated with not achieving the PASS, though meaningful clinical improvements still occurred in this group, and the majority were satisfied with their outcome. Importantly, a lower preoperative OHS should not be interpreted as a contraindication to surgery or used as a criterion for patient selection, as these patients still experienced clinically meaningful improvements exceeding the minimal clinically important difference and derived substantial benefit from THA despite being less likely to achieve the PASS threshold.

## Figures and Tables

**Table 1:** Demographics and preoperative patient-reported outcome measures according to whether a patient acceptable symptom state (PASS) in the Oxford Hip Score (OHS) was achieved at 12 months following total hip arthroplasty.

Demographic	Descriptive	PASS Achieved in OHS		Odds Ratio (OR) or Mean Difference (MD) (95% CI)	*p*-Value
		Yes (n = 3002)	No (n = 995)		
**Gender** (n, % of group)	Male	1263 (42.1)	359 (36.1)	OR 0.78	0.001 *
	Female	1739 (57.9)	636 (63.9)	(0.67 to 0.90)	
**Age** (years: mean, SD)		68.3 (11.4)	68.2 (12.4)	MD 0.1 (−0.9 to 1.9)	0.746 **
**BMI** (kg/m^2^: mean, SD)		27.9 (5.3)	29.7 (6.4)	MD 1.8 (1.3 to 2.2)	<0.001 **
**OHS** (mean, SD)		22.2 (8.9)	16.6 (8.0)	MD 5.6 (5.0 to 6.2)	<0.001 **
**EQ-5D** (mean, SD)		0.438 (0.328)	0.250 (0.342)	MD 0.187 (0.163 to 0.211)	<0.001 **
**EQ-VAS** (mean, SD)		70.6 (20.1)	59.5 (22.2)	MD 11.1 (9.6 to 12.6)	<0.001 **
**Pain VAS** (mean, SD)		52.8 (23.3)	48.1 (23.6)	MD 4.6 (2.9 to 6.4)	<0.001 **

* Chi-square test, ** Student’s independent *t*-test, SD: standard deviation, CI: confidence intervals.

**Table 2:** Logistic regression analysis of preoperative factors associated with achieving a patient acceptable symptom state (PASS) in the Oxford Hip Score (OHS) at 12 months following total hip arthroplasty. The model was statistically significant (chi-square test = 337.5, *p* < 0.001), indicating its ability to distinguish between patients who did and did not achieve a 12-month PASS in the OHS. The model as a whole explained between 11% (Cox and Snell R-squared) and 16% (Nagelkerke R-squared) of the variance in the pain status and correctly classified 95.9% of patients who achieved the PASS. Hosmer and Lemeshow test was not significant (*p* = 0.213), supporting the goodness of fit.

Preoperative Variables Included in Model		Odds Ratio (95% CI)	*p*-Value
**Gender**	Male	Reference	
	Female	0.85 (0.71 to 1.02)	0.074
**Age**		1.00 (0.99 to 1.01)	0.534
**BMI**		0.96 (0.95 to 0.98)	<0.001
**OHS**		1.05 (1.03 to 1.06)	<0.001
**EQ-5D**		1.88 (1.33 to 2.65)	<0.001
**EQ-VAS**		1.02 (1.01 to 1.02)	<0.001
**Pain VAS**		1.00 (0.99 to 1.00)	0.083

**Table 3:** Postoperative Oxford Hip Score (OHS), EQ-5D, EQ-VAS and pain VAS and the changes relative to baseline preoperative scores according to whether the patient acceptable symptom state (PASS) in the OHS was achieved at 12 months following total hip arthroplasty. The rows demonstrate the differences, while the columns demonstrate the comparison of the preoperative to 12-month postoperative outcomes within each of the groups (yes and no).

PROM and Timepoint	PASS Achieved in OHS		Difference (95% CI)	*p*-Value *
	Yes (n = 3002)	No (n = 995)		
**12-month OHS**	44.0 (3.6)	26.2 (7.4)	17.8 (17.5 to 18.2)	<0.001
**Change**	21.8 (8.9)	9.6 (9.1)	12.2 (11.5 to 12.8)	<0.001
**95% CI**	21.5 to 22.1	9.0 to 10.2		
***p*** -value **	<0.001	<0.001		
**12-month EQ-5D**	0.881 (0.152)	0.507 (0.286)	0.375 (0.361 to 0.389)	<0.001
**Change**	0.445 (0.341)	0.254 (0.388)	0.191 (0.165 to 0.217)	<0.001
**95% CI**	0.432 to 0.457	0.229 to 0.278		
***p*** -value **	<0.001	<0.001		
**12-month EQ-VAS**	83.5 (15.5)	60.8 (21.2)	22.7 (21.4 to 23.9)	<0.001
**Change**	12.8 (22.0)	1.4 (25.9)	11.4 (9.7 to 13.2)	<0.001
**95% CI**	12.0 to 13.6	−0.3 to 3.1		
***p*** -value **	<0.001	0.111		
**12-month Pain VAS**	83.1 (25.3)	56.7 (24.4)	26.4 (24.6 to 28.2)	<0.001
**Change**	30.4 (33.8)	8.2 (32.7)	22.2 (19.6 to 24.7)	<0.001
**95% CI**	29.1 to 31.7	6.1 to 10.3		
***p*** -value **	<0.001	<0.001		

* Independent Student’s *t*-test, ** paired Student’s *t*-test.

**Table 4:** Satisfaction with hip arthroplasty 12-months postoperatively according to whether the patient acceptable symptom state (PASS) in the Oxford Hip Score (OHS) was achieved at 12 months.

Satisfaction at 12 Months	PASS Achieved in OHS (n, %)		Odds Ratio (95% CI)	*p*-Value *
	Yes	No		
**Satisfied**	2930 (97.6)	704 (70.8)	16.8 (12.8 to 22.1)	<0.001
**Dissatisfied**	72 (2.4)	291 (29.2)		
**Total**	3002	995		

* Chi-square test.

**Table 5:** Demographics and preoperative patient-reported outcome measures according to patient satisfaction at 12 months for those not achieving a patient acceptable symptom state (PASS) in the Oxford Hip Score (OHS) at 12 months following total hip arthroplasty.

Demographic	Descriptive	Satisfied		Odds Ratio (OR) or Mean Difference (MD) (95% CI)	*p*-Value
		Yes (n = 704)	No (n = 291)		
**Gender** (n, % of group)	Male	255 (36.2)	104 (35.7)	OR 0.98	0.885 *
	Female	449 (63.8)	187 (64.3)	(0.74 to 1.300	
**Age** (years: mean, SD)		68.7 (12.2)	67.5 (12.6)	MD 1.2 (−0.5 to 2.9)	0.174 **
**BMI** (kg/m^2^: mean, SD)		29.9 (6.2)	29.3 (7.9)	MD 0.5 (−0.4 to 1.6)	0.220 **
**OHS** (mean, SD)		16.1 (8.0)	17.9 (7.9)	MD 1.8 (0.7 to 2.9)	0.002 **
**EQ-5D** (mean, SD)		0.240 (0.343)	0.277 (0.342)	MD 0.037 (−0.010 to 0.085)	0.124 **
**EQ-VAS** (mean, SD)		59.0 (22.0)	60.7 (21.7)	MD 1.1 (−1.4 to 4.8)	0.280 **
**Pain VAS** (mean, SD)		47.7 (24.2)	49.2 (22.4)	MD 1.5 (−1.9 to 4.8)	0.394 **

* Chi-square test, ** Student’s independent *t*-test, SD: standard deviation, CI: confidence interval.

## Data Availability

The raw data supporting the conclusions of this article will be made available by the authors on request.

## References

[1] Learmonth I.D., Young C., Rorabeck C. (2007). The operation of the century: Total hip replacement. Lancet.

[2] Ahmad M.A., Xypnitos F.N., Giannoudis P.V. (2011). Measuring hip outcomes: Common scales and checklists. Injury.

[3] Reiter C.R., Abraham V.M., Riddle D.L., Patel N.K., Goldman A.H. (2024). Patient reported outcome measures (PROMs) as primary and secondary outcomes in total hip and knee arthroplasty randomized controlled trials: A systematic review. Arch. Orthop. Trauma. Surg..

[4] Dawson J., Fitzpatrick R., Carr A., Murray D. (1996). Questionnaire on the perceptions of patients about total hip replacement. J. Bone Jt. Surg. Ser. B.

[5] Murray D.W., Fitzpatrick R., Rogers K., Pandit H., Beard D.J., Carr A.J., Dawson J. (2007). The use of the Oxford hip and knee scores. J. Bone Jt. Surg. Ser. B.

[6] Beard D.J., Harris K., Dawson J., Doll H., Murray D.W., Carr A.J., Price A.J. (2015). Meaningful changes for the Oxford hip and knee scores after joint replacement surgery. J. Clin. Epidemiol..

[7] Keurentjes J.C., Van Tol F.R., Fiocco M., So-Osman C., Onstenk R., Koopman-Van Gemert A.W.M.M., Pöll R.G., Nelissen R.G.H.H. (2014). Patient acceptable symptom states after total hip or knee replacement at mid-term follow-up. Bone Jt. Res..

[8] Pozzuoli A., Berizzi A., Crimì A., Belluzzi E., Frigo A.C., Conti G.D., Nicolli A., Trevisan A., Biz C., Ruggieri P. (2020). Metal Ion Release, Clinical and Radiological Outcomes in Large Diameter Metal-on-Metal Total Hip Arthroplasty at Long-Term Follow-Up. Diagnostics.

[9] Harris L.K., Troelsen A., Terluin B., Gromov K., Overgaard S., Price A., Ingelsrud L.H. (2023). Interpretation Threshold Values for the Oxford Hip Score in Patients Undergoing Total Hip Arthroplasty: Advancing Their Clinical Use. J. Bone Jt. Surg. Am..

[10] Geilen J.E.J.W., Hoelen T.A., Schotanus M.G.M., van Hemert W.L.W., Spekenbrink-Spooren A., Boonen B., Most J. (2025). Defining Clinically Meaningful Thresholds for 12-Month Patient-Reported Outcomes in Total Hip Arthroplasty; Toward Improving Threshold Accuracy. Arthroplast. Today.

[11] Abugu G.U., Holloway N., Riches P., Clarke J., Giardini M.E., Chopra S. (2026). Weighted predictive modelling estimation of patient acceptable symptom state for forgotten joint score, Oxford hip score, and EuroQol health index 3 and 12 months after total hip arthroplasty in a United Kingdom cohort. Qual. Life Res..

[12] Longo U.G., De Salvatore S., Piergentili I., Indiveri A., Di Naro C., Santamaria G., Marchetti A., Marinis M.G., Denaro V. (2021). Total Hip Arthroplasty: Minimal Clinically Important Difference and Patient Acceptable Symptom State for the Forgotten Joint Score 12. Int. J. Environ. Res. Public Health.

[13] Galea V.P., Ingelsrud L.H., Florissi I., Shin D., Bragdon C.R., Malchau H., Gromov K., Troelsen A. (2020). Patient-acceptable symptom state for the Oxford Hip Score and Forgotten Joint Score at 3 months, 1 year, and 2 years following total hip arthroplasty: A registry-based study of 597 cases. Acta Orthop..

[14] Zhang Y., Xi X., Huang Y. (2023). The anchor design of anchor-based method to determine the minimal clinically important difference: A systematic review. Health Qual. Life Outcomes.

[15] Wylde V., Learmonth I.D., Cavendish V.J. (2005). The Oxford hip score: The patient’s perspective. Health Qual. Life Outcomes.

[16] Henry P., Blandeau M., Pudlo P., Wallard L. (2026). Surgical approach and functional recovery after total hip arthroplasty within the International Classification of Functioning model. Ann. Phys. Rehabil. Med..

[17] Kawai T., Kataoka M., Goto K., Kuroda Y., So K., Matsuda S. (2018). Patient- and Surgery-Related Factors that Affect Patient-Reported Outcomes after Total Hip Arthroplasty. J. Clin. Med..

[18] Aggarwal A., Naylor J.M., Adie S., Liu V.K., Harris I.A. (2022). Preoperative Factors and Patient-Reported Outcomes After Total Hip Arthroplasty: Multivariable Prediction Modeling. J. Arthroplast..

[19] Judge A., Batra R.N., Thomas G.E., Beard D., Javaid M.K., Murray D.W., Dieppe P.A., Dreinhoefer K.E., Peter-Guenther K., Field R. (2014). Body mass index is not a clinically meaningful predictor of patient reported outcomes of primary hip replacement surgery: Prospective cohort study. Osteoarthr. Cartil..

[20] Vijayananthan A., Nawawi O. (2008). The importance of Good Clinical Practice guidelines and its role in clinical trials. Biomed. Imaging Interv. J..

[21] Makaram N., Lee T., Macdonald D., Clement N.D. (2020). The verbal Oxford Knee Score is not clinically different from the written score when assessed before or after total knee arthroplasty. Knee.

[22] Dawson J., Fitzpatrick R., Murray D., Carr A. (1998). Questionnaire on the perceptions of patients about total knee replacement. J. Bone Jt. Surg. Br..

[23] Brooks R. (1996). EuroQol: The current state of play. Health Policy.

[24] Clement N.D., MacDonald D., Simpson A.H. (2014). The minimal clinically important difference in the Oxford knee score and Short Form 12 score after total knee arthroplasty. Knee Surg. Sports Traumatol. Arthrosc..

[25] Yapp L.Z., Scott C.E.H., Howie C.R., MacDonald D.J., Simpson A.H.R.W., Clement N.D. (2022). Meaningful values of the EQ-5D-3L in patients undergoing primary knee arthroplasty. Bone Jt. Res..

[26] Scott C.E., Howie C.R., MacDonald D., Biant L.C. (2010). Predicting dissatisfaction following total knee replacement: A prospective study of 1217 patients. J. Bone Jt. Surg. Br..

[27] Mandrekar J.N. (2010). Receiver operating characteristic curve in diagnostic test assessment. J. Thorac. Oncol..

[28] Gwynne-Jones D.P., Sullivan T., Wilson R., Abbott J.H. (2020). The Relationship Between Preoperative Oxford Hip and Knee Score and Change in Health-Related Quality of Life After Total Hip and Total Knee Arthroplasty: Can It Help Inform Rationing Decisions?. Arthroplast. Today.

[29] Clement N.D., Wickramasinghe N.R., Bayram J.M., Hughes K., Oag E., Heinz N., Fraser E., Jefferies J.G., Dall G.F., Ballantyne A. (2022). Significant deterioration in quality of life and increased frailty in patients waiting more than six months for total hip or knee arthroplasty: A cross-sectional multicentre study. Bone Jt. J..

[30] Holtzman J., Saleh K., Kane R. (2002). Gender differences in functional status and pain in a Medicare population undergoing elective total hip arthroplasty. Med. Care.

[31] Warnock J.M., Karayiannis P.N., Gallagher N.E., Hill J.C., Beverland D.E. (2020). Are There Gender-Specific Errors in Restoration of Hip Biomechanics That Affect Outcome Following Total Hip Arthroplasty?. J. Arthroplast..

[32] Benyamini B., Emara A.K., Pasqualini I., Ibaseta A., Klika A.K., Khan S.T., Zielinski M.R., Adult Reconstruction Research C.C., Piuzzi N.S. (2025). Mapping the importance of each individual element accounted by HOOS and VR-12 on 1-year patient satisfaction after primary total hip arthroplasty: A prospective institutional analysis. Eur. J. Orthop. Surg. Traumatol..

[33] Rossi N., Nannini A., Ulivi M., Sirtori P., Banfi G., Tomaiuolo R., de Girolamo L., Mangiavini L., Peretti G.M. (2024). Men and women undergoing total hip arthroplasty have different clinical presentations before surgery and different outcomes at 1-year follow-up. Knee Surg. Sports Traumatol. Arthrosc..

[34] Lovie J., Clement N.D., MacDonald D., Ahmed I. (2023). Diabesity: A superadded effect contributing to worse total primary hip replacement operative outcomes for patients with diabetes and obesity. Arch. Orthop. Trauma. Surg..

[35] Huffman N., Pasqualini I., Redfern R.E., Murray T.G., Deren M.E., Israelite C.L., Nelson C.L., Van Andel D., Cholewa J.M., Anderson M.B. (2024). Patient satisfaction and patient-reported outcomes do not vary by BMI class in total hip arthroplasty. Eur. J. Orthop. Surg. Traumatol..

[36] Kuklinski D., Marques C.J., Bohlen K., Westphal K.C., Lampe F., Geissler A. (2022). Thresholds for meaningful improvement in WOMAC scores need to be adjusted to patient characteristics after hip and knee replacement. J. Orthop..

[37] Luger M., Schopper C., Krottenthaler E.S., Mahmoud M., Heyse T., Gotterbarm T., Klasan A. (2023). Not all questions are created equal: The weight of the Oxford Knee Scores questions in a multicentric validation study. J. Orthop. Traumatol..

[38] Hooper G.J., Rothwell A.G., Hooper N.M., Frampton C. (2012). The relationship between the American Society of Anesthesiologists physical rating and outcome following total hip and knee arthroplasty: An analysis of theNew Zealand Joint Registry. J. Bone Jt. Surg. Am..

[39] Shah V.I., Pachore J.A., Upadhyay S., Shah K., Seth A., Kshatriya A., Patil J., Gujjar P., Kantesariya M. (2021). Predictors of 90-Day All-Cause Morbidity, Mortality and Poor Functional Outcome Scores Following Elective Total Knee Arthroplasty in a High-Volume Setting: A Prospective Cohort Study. Indian. J. Orthop..

[40] Teni F.S., Burström K., Berg J., Leidl R., Rolfson O. (2020). Predictive ability of the American Society of Anaesthesiologists physical status classification system on health-related quality of life of patients after total hip replacement: Comparisons across eight EQ-5D-3L value sets. BMC Musculoskelet. Disord..

